# Spherified Pd_0.33_Ni_0.67_/BCNT Catalyst for Nitrobenzene Hydrogenation

**DOI:** 10.3390/ijms26115420

**Published:** 2025-06-05

**Authors:** Csenge Nagy, Emőke Sikora, Ádám Prekob, Kitti Gráczer, Gábor Muránszky, László Vanyorek, Ferenc Kristály, Zsolt Fejes

**Affiliations:** 1 Higher Education and Industrial Cooperation Centre, University of Miskolc, Miskolc-Egyetemváros, 3515 Miskolc, Hungary; csenge.nagy1@uni-miskolc.hu (C.N.); kitti.graczer@uni-miskolc.hu (K.G.); 2 Institute of Chemistry, University of Miskolc, Miskolc-Egyetemváros, 3515 Miskolc, Hungary; emoke.sikora@uni-miskolc.hu (E.S.); gabor.muranszky@uni-miskolc.hu (G.M.); laszlo.vanyorek@uni-miskolc.hu (L.V.); zsolt.fejes@uni-miskolc.hu (Z.F.); 3 Institute of Mineralogy and Geology, University of Miskolc, Miskolc-Egyetemváros, 3515 Miskolc, Hungary; askkf@uni-miskolc.hu

**Keywords:** carbon nanotubes, nitrobenzene, hydrogenation, catalyst, solvent

## Abstract

A separable bamboo-like carbon nanotube-based catalyst was prepared by the spherfication method using sodium alginate and nickel. The spheres were carbonized and then decorated with palladium nanoparticles before they were tested in nitrobenzene hydrogenation. The test was repeated with five different commonly used solvents (methanol, ethanol, isopropanol, tetrahydrofuran, and acetonitrile). According to the results, polar solvents showed a significantly higher aniline yield than the more apolar solvents and exceptional results were reported for ethanol (~100%). The catalyst was reused two more times (four hours each) to check the Pd leaching where the spheres kept their shape (despite the high mechanical friction caused by the mixer) and only a relatively low Pd amount was lost (5.48 rel.%). The catalyst was easily retrievable.

## 1. Introduction

The synthesis of aniline plays a significant role in the chemical industry, as it is the initial step in the production of MDI (methylene diphenyl diisocyanate). In acidic media, aniline and formaldehyde react to form MDA (methylene diphenyl diamine), which is then used to produce MDI through phosgenation. According to the 2023 data, one of the main producers manufactures aniline at a rate of 200 kt/year and MDI at 400 kt/year. MDI is an essential raw material for polyurethane foams, which are utilized in the construction and packaging industries [1,2].

The hydrogenation of nitrobenzene is the most common technology used to produce aniline. The reaction mechanism usually follows the Haber mechanism, which consists of a direct and a condensation pathway. The direct pathway can be divided into three steps. First, the reactants form nitrosobenzene and *N*-phenylhydroxylamine. Further hydrogenation converts the hydroxylamine intermediate into aniline. In the condensation reaction pathway, the condensation of the two previously mentioned intermediates leads to the formation of azoxybenzene, azobenzene, or hydrazobenzene, which are subsequently hydrogenated to aniline [3,4].

The liquid-phase hydrogenation of nitrobenzene can be carried out in various solvents, such as methanol, ethanol, isopropanol, glycerol, ethylene glycol, tetrahydrofuran, acetone, ethyl acetate, acetonitrile, dichloromethane, benzene, hexane, aniline and acidic solutions such as acetic acid or even in water [5,6]. Based on previous research in which apolar solvents were used, the conversion of nitrobenzene is significantly lower compared to polar solvents, such as methanol or ethanol [5,6,7,8,9,10,11].

The hydrogenation usually takes place with the application of Pd or Pt catalysts with different types of carbon carriers, such as activated carbon (AC), carbon black (CB) [12], or carbon nanotubes (CNTs) [13,14,15]. Carbon nanotubes are widely used in catalytic reactions as a catalyst carrier mostly because of their high specific surface area and structural properties [16,17,18,19,20,21]. The structural characteristics of carbon nanotubes can be modified for instance by incorporation of heteroatoms (such as N, B, or P), or doping. During N-doping, nitrogen is incorporated into the hexagonal lattice, forming bonds with carbon atoms providing a structure similar to the bamboo (bamboo-like carbon nanotubes, BCNT). The incorporation of nitrogen atoms distorts the regular crystal lattice structure, leading to vacancies in the CNT structure. These nitrogen atoms can be pyrrolic, pyridinic, pyrazolic, or graphitic, depending on their location. The pyrrolic nitrogen on the BCNT surface functions as a catalytically active site, significantly promoting the chemisorption and dissociation of hydrogen molecules since the pyrrolic complexes are more reactive than the other forms according to calculations [22]. Nitrogen atoms can also form bonds with the adsorbate, which enhances the stability and activity of the catalyst [20,23,24,25,26,27].

However, the separation of powder catalysts like activated carbon or carbon nanotubes can be an expensive and time-consuming process using either filters with low pore diameters or sedimentation; therefore, the use of formulated or template--based catalysts could be advantageous. “Spherification” is a process where caviar-like spheres can be prepared by using sodium alginate, making it possible to form spheres from a powder, thereby enhancing its separability from liquids. By carbonizing the spheres, carbon-based catalyst supports with a few millimeters in diameters can be produced. Since the carbon nanotubes are known for their exceptional mechanical properties, they provide high mechanical resistance for the prepared spheres, meaning that it is also possible to use them in systems equipped with a mixer.

In this paper, the spherification method was used to prepare a BCNT-based catalyst with enhanced recoverability. The prepared Pd_0.33_Ni_0.67_/BCNT catalyst was tested in multiple cycles in nitrobenzene hydrogenation using five different solvents.

## 2. Results and Discussion

### 2.1. Results of the Characterization

**Figure 1:**
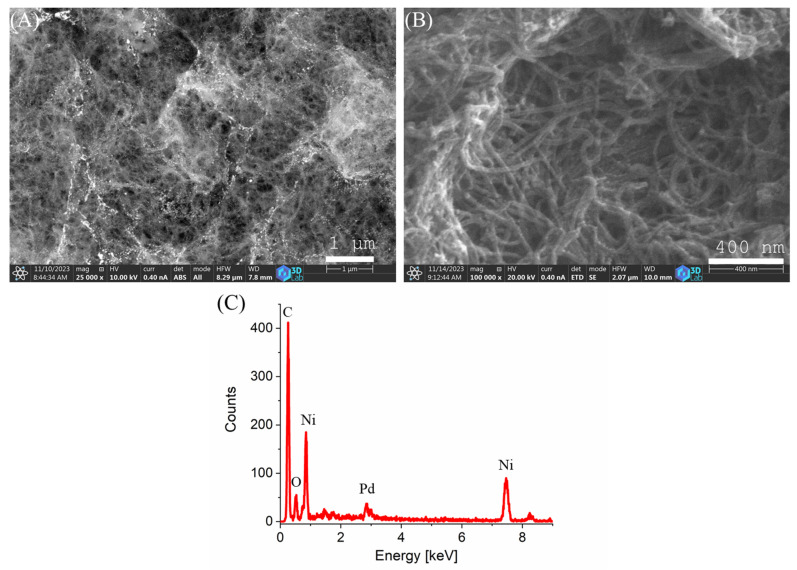
SEM images (**A**,**B**) at different magnifications and EDS map (**C**) of the catalyst.

The morphology of the prepared catalyst was examined using scanning electron microscopy (SEM). SEM images taken at two different magnifications and detectors are presented in Figure 1. In Figure 1A, on the surface of the carbon support, glowing white Pd nanoparticles can be seen. In higher magnification (Figure 1B), the characteristic dense fibrous structure of the carbon nanotubes, interconnected and stabilized by a carbonized alginate framework, is visible. Elemental analysis was also performed by energy-dispersive X-ray spectroscopy (EDS) (Figure 1C). The analysis revealed characteristic peaks associated with the expected elements: carbon (C), oxygen (O), palladium (Pd), and nickel (Ni).

Textural properties of the Pd_0.33_Ni_0.67_/BCNT catalyst, including specific surface area and average pore diameter, were determined by nitrogen adsorption–desorption measurements using the Brunauer–Emmett–Teller (BET) method. The specific surface area of the catalyst was found to be 118.4 m^2^/g, and the average pore diameter was 19.9 nm. The isotherm of both the support and the catalyst showed significant mesoporosity (Figure S1). Typically, nitrogen-doped carbon nanotubes exhibit specific surface areas ranging from 200 to 700 m^2^/g, with an average pore diameter between 2 and 20 nm. For comparison, single-walled carbon nanotubes (SWCNTs) usually have surface areas between 400 and 900 m^2^/g, while multi-walled carbon nanotubes (MWCNTs) show values in the range of 200–400 m^2^/g [18,21]. In our case, the lower specific surface area may be attributed to the spherification of the BCNT-containing support.

The metal content of the catalyst was determined by inductively coupled plasma optical emission spectroscopy (ICP-OES). The palladium loading on the catalyst was 2.92 wt%. The relatively high nickel content (22.77 wt%) can be attributed to the spherification-based preparation method where Ni is introduced into the sample via ion exchange processes and also from the residual Ni from the catalyst used in the synthesis of the carbon nanotubes itself.

**Figure 2:**
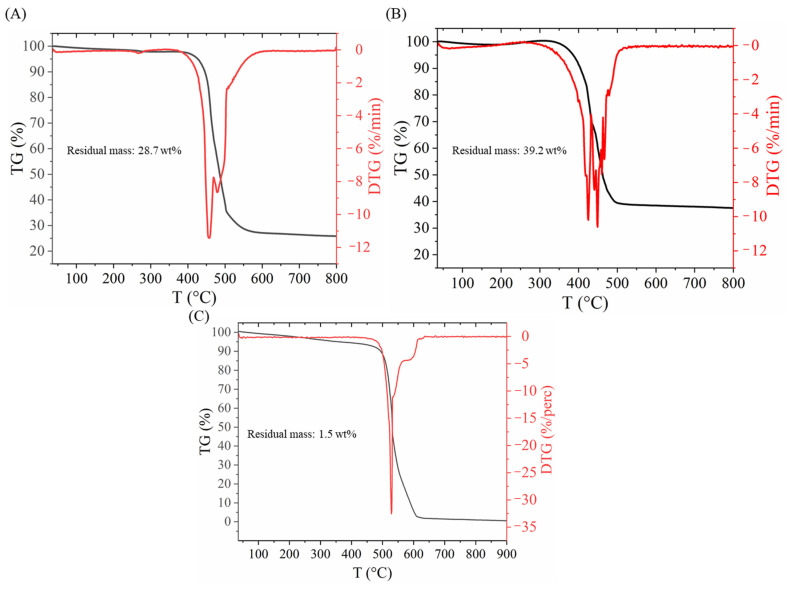
Thermogravimetric analysis of Ni/BCNT (**A**), Pd_0.33_Ni_0.67_/BCNT (**B**) and BCNT (**C**) samples.

To check the whole metal content as a control, thermogravimetric analysis was also carried out (Figure 2). The residual mass of the support was 28.7 wt%, while in the case of the catalyst, it was 39.2%. It can also be seen that the decomposition of carbon starts earlier in the case of the catalyst, which can be explained with the catalytic effect of the Pd (Figure 2A,B). The shape of the TG curve shows the typical decomposition of a BCNT sample (Figure 2C). The wider decomposition temperature range also indicates the presence of different carbon forms with a different structural order and amount of defects presumably from the decomposition of the sodium alginate.

**Figure 3:**
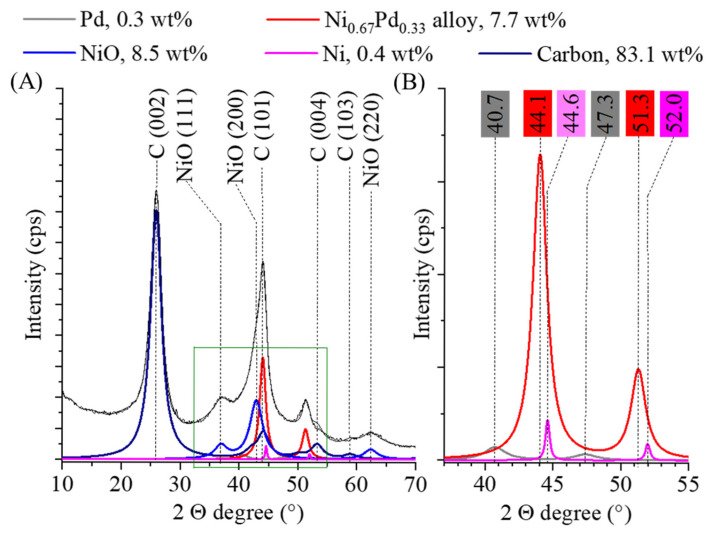
Rietveld refined X-ray diffractogram of the Pd_0.33_Ni_0.67_/BCNT catalyst (**A**) and a magnified section of it with typical reflections of nickel, palladium and the alloy phase (**B**).

In order to confirm the presence of catalytically active phases in the sample, X-ray diffraction measurements were performed. Reflections indicating the presence of carbon are visible at 25.9° (002), 43.9° (101), 53.4° (004) and 58.8° (103) 2 ϴ degrees (PDF 75–1621) (Figure 3A). The carbon in the substrate is mostly found in the form of carbon nanotubes, but carbon from the decomposition of alginate is also present, with a combined amount of 83.1 wt%. During gel formation from the sodium alginate, the sodium ions were replaced by nickel (II) ions in the polymer chain and nickel oxide was also formed after carbonization, with a content of 8.5 wt%. Peaks of the NiO phase can be found at 36.9° (111), 42.9° (200) and 624 (220) 2 Θ degrees (PDF 47–1049). Nickel was also present in the sample in the amount of 0.4 wt% due to hydrogenation. The characteristic reflections of Ni can be found at 44.6° (111) and 52.0° (200) two theta degrees (PDF 04–0850) (Figure 3B). The average crystallite size of the nickel domains was 4 ± 1 nm. Moreover, on the diffractogram, the reflections of the face-centered cubic crystalline (fcc) palladium structure can be identified at 40.7° (111) and 47.3° (200) two theta degrees and their crystallite size was 5 ± 2 nm (Figure 3B) (PDF 046–1043). The amount of elemental palladium was 0.3 wt%. The low amount of elemental nickel and palladium can be explained by the formation of the nickel–palladium alloy phase, as evidenced by the reflection at 44.1° and 51.3° two theta degrees (Figure 3B). The sign of alloy forming is that the main peaks of the nickel (III) at 44.6° and (200) at 52.0°) shift to lower angles (44.1°and 51.3°) since the lattice parameters change [28]. The nickel to palladium ratio in the alloy phase was 0.67:0.33 and the average particle size was very similar than the elemental palladium and nickel crystallites (5 ± 1 nm). The amount of alloy in the catalyst was 7.7 wt%. The formation of the alloy phase leads to electron transfer processes that contribute significantly to the increase in catalytic activity since the Ni donates electrons for the Pd, thereby weakening its H* adsorption. The application of alloys proved to be advantageous in many different cases before [29,30,31,32]. González-Vera et al. prepared a decorated Pd-Ni alloy TiO_2_ catalyst for nitrobenzene hydrogenation and proved that the use of the alloy phase increased the operational stability and the aniline selectivity [33].

**Figure 4:**
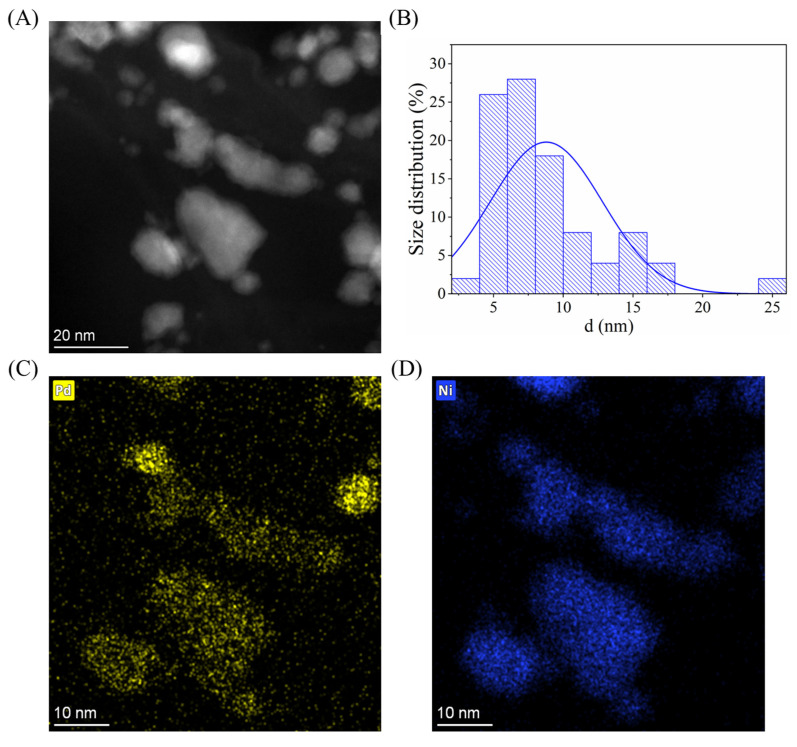
HAADF STEM image of Pd_0.33_Ni_0.67_/BCNT catalyst ((**A**), 1,100,000× magnification), size distribution of the particles on the BCNT surface (**B**), and the element maps of palladium (**C**) and nickel (**D**).

To confirm the alloy phase and determine the particle size and distribution, TEM image (Figure 4A) and element maps were taken (Figure 4C,D). For the size determination, 100 nanoparticles were measured using ImageJ (v1.54p) image analysis software on multiple TEM images. It is visible that the Pd and Ni particles cannot be separated and they form single particles with a bigger size (Figure 4C,D). This fact correlates well with the result of the XRD measurement and the Pd and Ni particles are mainly present in alloy form. Since both elements are present in the visible particles, the measured size distribution shows the size of the particle mixture of Pd, Ni, NiO, and Ni_0.67_Pd_0.33_. (Figure 4B). It can be seen that the majority of the particles are smaller than 20 nm, while the average diameter is 9 ± 4 nm. On the TEM image and element maps with lower magnification, it is visible that the particles cover the BCNTs well (Figure S2).

### 2.2. Catalytic Results

**Figure 5:**
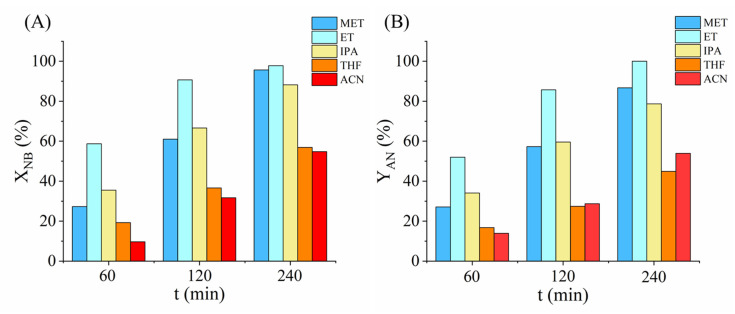
Nitrobenzene conversion (**A**) and aniline yield (**B**) after 60, 120 and 240 min of hydrogenation in five different solvents.

Solvent polarity plays a crucial role in hydrogenation reactions. In this paper, we tested the prepared catalyst in five different nitrobenzene (NB) solutions using common solvents: methanol (MET), ethanol (ET), isopropanol (IPA), tetrahydrofuran (THF), and acetonitrile (ACN). We used the same test parameters for all the measurements (50 °C, 20 barg, 5.14 mol NB/g Pd). In case of nitrobenzene hydrogenation, the main product is aniline (AN), which is produced by hydrogenolysis of the –NO_2_ group. Since polar protic solvents can better stabilize the transition states through hydrogen bonding network than aprotic ones, they can enhance both the reaction rate and the selectivity to aniline. It is also confirmed by our results (Figure 5), in which the alcohols gave significantly higher aniline yield than the aprotic solvents. However, comparing the alcohols, ethanol provided the highest product yield with excellent selectivity (97.8% NB conversion, 99.9% AN selectivity) than the more polar methanol. Therefore, solvent polarity itself cannot explain the results. The performance of the catalyst was compared to other conventional catalysts and also with one of our earlier studied catalysts (Table S1).

Since we used 96% (*v*/*v*) ethanol, we suspected that the 4% (*v*/*v*) water content further enhanced the reaction rate since it further increases the solvent polarity, which is advantageous in terms of reaction rate because less polar solvent molecules compete for the catalyst adsorption sites [34]. To check this assumption, we also ran a test in absolute ethanol to see the difference. However, the application of absolute ethanol further increased the aniline by about 7%, so the exceptional result is due to the ethanol itself (Figure S3). Hydrogen solubility is another important factor, since a higher hydrogen concentration can increase the chance of the reaction with the substrate molecules. d’Angelo et al. measured the hydrogen solubility of four alcohols (methanol, ethanol, propanol, and butanol), and stated that it increases with the chain length of the alcohol [35]. However, according to their results, ethanol showed an exception since its hydrogen dissolving capacity was almost as high as butanol compared to its expected capacity considering its place in the order. It is very likely that the 4% (*v*/*v*) water decreased the reaction rate by its lower hydrogen dissolving capacity.

Regarding the rate determining reaction step, Kochetova et al. [36] calculated that in different solvents, the rate determining step differs. According to their results, in ethanol, the hydrogenation of nitrobenzene determines the reaction rate, but in isopropanol, the reduction of nitrozobenzene demonstrates the lowest rate. Since at the beginning of the reaction, a high number of nitrobenzene molecules are present without any nitrozobenzene, ethanol has a more pronounced rate-enhancing effect [36].

The support (Ni-BCNT) was also tested without any Pd on the surface but no catalytic activity was detected. The catalytic activity is only attributed to mainly the Pd_0.33_Ni_0.67_ alloy and the Pd nanoparticles with a smaller percentage.

The catalytic tests were repeated with the already used catalyst two more times, without any washing or reactivating steps, to check Pd leaching and the catalytic activity in all of the solvents (Table 1). After the third run, the aniline yield decreased by about 25%, 32%, and 39% in the alcoholic solvents, in THF, and in ACN, respectively. The rate of Pd loss is usually the highest after the first cycle where the weakly adsorbed particles leave the surface. Therefore, the differences in terms of NB conversion and AN yield between the first and second cycles are usually much more significant than between the second and the third run. This could also be attributed to the various contamination of the catalyst surface caused by the poorly desorbing reaction components (e.g., side products), which in turn decrease and/or deactivate the catalyst surface.

Using ICP-OES, we measured the Pd content of all the used catalysts and determined an average of 2.76 wt% Pd, which means that only 5.48 ± 0.2% of the Pd leached from the initial loading (2.92 wt%). By using fixed bed reactors, which prevents the catalysts from facing the high mechanical abrasion of the mixer, the already low Pd leach could probably be further decreased.

## 3. Methods and Materials

### 3.1. Materials

For the spherification, sodium alginate (NaC_6_H_7_O_6_, Sigma Aldrich, St. Louis, MO, USA) and nickel (II) nitrate hexahydrate ((Ni(NO_3_))_2_ · 6 H_2_O, Sigma Aldrich) were used. The spheres were carbonized in nitrogen atmosphere (N_2_, 4.0, SIAD) and then decorated with Pd nanoparticles using palladium nitrate dihydrate (Pd (NO_3_)_2_ 2 · H_2_O, Alfa Aesar, Heysham, UK). The catalyst was activated in hydrogen atmosphere (H_2_, 4.5, SIAD, Bergamo, Italy). For the catalytic tests, nitrobenzene (C_6_H_5_NO_2_, 99.5%, Thermo Scientific) was used as a reactant, *p*-xylene (C_8_H_10_, GPR Rectapur, VWR Chemicals, Radnor, PA, USA) as a standard for gas chromatography and hydrogen gas (H_2_, 5.0, SIAD) and five different solvents, including methanol (CH_3_OH, technical, Molar Chemicals, Halásztelek, Hungary), ethanol (C_2_H_5_OH, 96% (v/v), VWR Chemicals), 2-propanol, (C_3_H_7_OH, 99.9%, Molar Chemicals), tetrahydrofuran (C_4_H_8_O, GPR Rectapur, VWR Chemicals) and acetonitrile (CH_3_CN, 99.9%, VWR Chemicals), were used.

### 3.2. Preparation of the Catalyst

**Figure 6:**
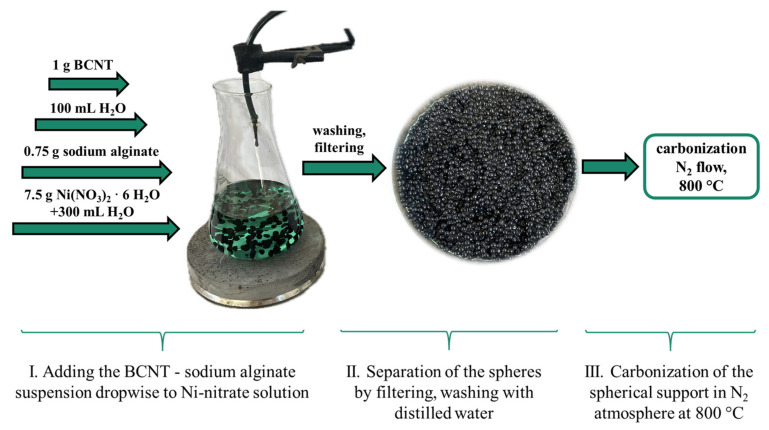
The preparation process of Ni/BCNT support by spherification method.

The bamboo-like carbon nanotube (BCNT) synthesis procedure followed the same methodology as described in our previous work [37]. The preparation of the spherified catalyst supports was based on a method also reported in our previous study [37]. A mixture of 100 mL deionized water, 0.750 g sodium alginate, and 1.000 g N-BCNT was prepared using a Hielscher homogenizer. This mixture was added dropwise to the solution of 7.500 g Ni(NO_3_)_2_ · 6 H_2_O in 300 mL deionized water (0.1368 molL^−1^ solution, pH 6.8) using a syringe pump (150 mL/h). After the addition, the solvent was removed by filtering and the spheres were washed with deionized water and dried at 105 °C for 24 h. The prepared beads were calcinated at 800 °C under a nitrogen flow for 60 min (Figure 6). Palladium was adsorbed by the supports by impregnation–reduction. A total of 1.150 g of the catalyst support was added to a 100 mL aqueous solution containing 0.100 g of Pd(NO_3_)_2_ 2 H_2_O followed by vacuum evaporation and reduction at 400–on in a H_2_ stream for 30 min.

### 3.3. Characterization Methods

The surface of the catalyst was examined by a Helios G4 PFIB CXe Plasma Focused Ion Beam Scanning Electron Microscope (PFIB-SEM, Thermo Fisher Scientific, Waltham, MA, USA) equipped with an EDAX Octane Elect EDS System and APEX Analysis Software (v5.6.1.). Carbon tape was used for sample preparation. To examine the catalytically active nanoparticles, HRTEM (Talos F200X G2, Thermo Scientific, Waltham, MA, USA) with a field emission electron gun named X-FEG (accelerating voltage: 20–200 kV) was used, according to the HAADF STEM (high-angle annular dark-field scanning transmission electron microscopy) method. The palladium and nickel content of the catalyst samples was determined by using a Varian 720 ES inductively coupled optical emission spectrometer (ICP-OES, Varian, Palo Alto, CA, USA). For the measurements, the samples were first burnt in a furnace at 600 °C for 1 h, cooled to room temperature and then dissolved in hydrochloric acid (36 wt%) and nitric acid (65 wt%). The residual mass was measured by the Trasus 209 thermogravimetric microbalance (TGA, NETZSCH, Selb, Bavaria, Germany). The specific surface area and pore volume of the catalyst was measured by N_2_ adsorption experiments using Micromeritics Tristar 3000 sorptometer (Micromeritics, Norcross, GA, USA). The calculations were carried out based on BET isotherms. To analyze the composition of the samples taken during the catalytic reaction, an Agilent 88 gas chromatograph (Agilent, Santa Carla, CA, USA) equipped with a flame ionization detector (FID) and an HP-5 capillary column (30 m × 0.320 mm × 0.25 μm) was used.

### 3.4. Catalytic Tests of the CaEquationtalyst in Nitrobenzene Hydrogenation

The hydrogenation of nitrobenzene was carried out in methanol (MET), ethanol (ET), isopropanol (IPA), tetrahydrofuran (THF), and acetonitrile (ACN). The concentration of nitrobenzene was 0.10 mol L^−1^. Next, 0.100 g of the catalyst was added to 150 mL of nitrobenzene solution. A Büchi Uster Picoclave reactor system equipped with a 200 mL stainless steel vessel and a heating jacket was used for the hydrogenation tests. We used 20 barg H_2_ pressure and 50 °C during all of the reactions. Samples were taken at 5, 10, 15, 20, 30, 40, 60, 80, 120, 180, and 240 min.

Based on the results of the gas chromatographic measurements, nitrobenzene (NB) conversion (X%) was calculated using the following equation (Equation (1)).

(1) $$X\%=\frac{\;n_{consumed\;nitrobenzene}}{n_{initial\;nitrobenzene}}\;\cdot \;100$$

The aniline (AN) yield (Y%) was also calculated as follows (Equation (2)).

(2) $$Y\%=\frac{{n\;}_{formed\;aniline}}{n_{\;theoritical\;aniline\;}}\;\cdot \;100$$

## 4. Conclusions

In this paper, a Pd- and Ni-containing carbon nanotube catalyst was prepared using the spherification method. As a result, a sphere-like carbon support with a few millimeters in diameter was prepared, which provided easy recoverability. The Pd was added by the impregnation method (2.92 wt%). Due to the excellent mechanical properties of the carbon nanotubes, the catalyst also resisted the mechanical abrasion caused by the mixer, ensuring that the catalyst can be used in multiple test cycles. The catalyst was tested in nitrobenzene hydrogenation with five different solvents (methanol, ethanol, isopropanol, tetrahydrofuran, and acetonitrile), where ethanol provided the highest aniline yield (99.9% in the first cycle) after four hours of reaction time. The exceptional result in ethanol could be explained by its high hydrogen solubility property. According to the XRD measurements, the Pd and Ni formed an alloy (due to the 400 °C heat treatment in hydrogen atmosphere), which provided excellent aniline selectivity and catalyst operation stability. When using the catalyst in multiple test cycles (four hours each), only an average of 5.48% of the initial Pd content was lost after three cycles.

## Figures and Tables

**Table 1:** Nitrobenzene conversion (*X*_NB_) and aniline yield (*Y*_AN_) in five different solvents at 60, 120, and 240 min in the three test cycles.

Solvent	Cycle	*X*_NB_	*Y*_AN_
		240 min	240 min
MET	1	95.7%	86.7%
	2	87.2%	83.1%
	3	74.5%	65.9%
ET	1	97.8%	99.9%
	2	85.7%	82.0%
	3	78.7%	72.1%
IPA	1	88.2%	78.7%
	2	59.6%	67.8%
	3	54.4%	43.2%
THF	1	56.9%	44.9%
	2	20.2%	15.5%
	3	21.8%	12.9%
ACN	1	54.8%	53.9%
	2	32.8%	25.9%
	3	22.4%	14.9%

## Data Availability

Data contained within the article.

## Supplementary Materials

The following supporting information can be downloaded at https://www.mdpi.com/article/10.3390/ijms26115420/s1, References [38,39,40,41] are cited in the Supplementary Materials.
