# Supplementary material for: Spherified Pd0.33Ni0.67/BCNT Catalyst for Nitrobenzene Hydrogenation
Source: Int J Mol Sci. 2025 Jun 5;26(11):5420. doi: 10.3390/ijms26115420 (PMC12155155; doi:10.3390/ijms26115420)
Supplement: Supplementary file 1 [file ijms-26-05420-s001.zip › ijms-3662180-supplementary.pdf]

Spherified $Pd_{0.33}Ni_{0.67}$/BCNT Catalyst for Nitrobenzene Hydrogenation

Csenge Nagy [1], Emőke Sikora [2], Ádám Prekob [2,*], Kitti Gráczer [1], Gábor Muránszky [2], László Vanyorek [2], Ferenc Kristály [3] and Zsolt Fejes [2]

[1]Higher Education and Industrial Cooperation Centre, University of Miskolc, Miskolc-Egyetemváros, 3515 Miskolc, Hungary; csenge.nagy1@uni-miskolc.hu (C.N.); kitti.graczer@uni-miskolc.hu (K.G.)
[2]Institute of Chemistry, University of Miskolc, Miskolc-Egyetemváros, 3515 Miskolc, Hungary; emoke.sikora@uni-miskolc.hu (E.S.); gabor.muranszky@uni-miskolc.hu (G.M.); laszlo.vanyorek@uni-miskolc.hu (L.V.); zsolt.fejes@uni-miskolc.hu (Z.F.)
[3] Institute of Mineralogy and Geology, University of Miskolc, Miskolc-Egyetemváros, 3515 Miskolc, Hungary; askkf@uni-miskolc.hu
*Correspondence: adam.prekob@uni-miskolc.hu

Specific surface area determination by BET method

The specific surface area was measured for both the Ni/BCNT support and the $Pd_{0.33}Ni_{0.67}$/BCNT catalyst. The surface area decreased from 133.8 m^2/g to 118.4 m^2/g after the Pd decoration step since during the impregnation method many pores get blocked therefore decreasing the surface area. The BET isotherms have a similar form in both cases indicating mesoporosity both before and after the Pd decoration (Figure S1).

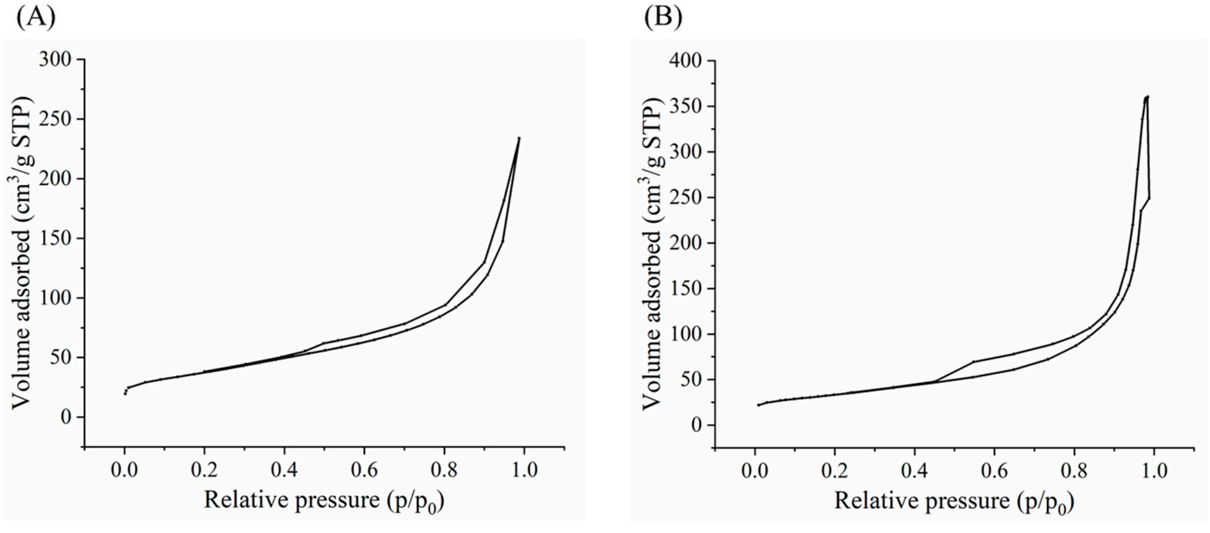

Figure S1: Whole isotherms of the Ni/BCNT support (A) and the $Pd_{0.33}Ni_{0.67}$/BCNT catalyst (B)

Transmission electron microscopy (TEM)

TEM images were taken with lower magnification to visualize the metal (and oxide) particles on the support surface (Figure S2). The element mapping shows that the Pd and Ni enrichments are at the same place further confirms the fact that an alloy was formed. It should be also mentioned that the BCNTs also contained nickel since they were prepared using nickel contained catalyst.

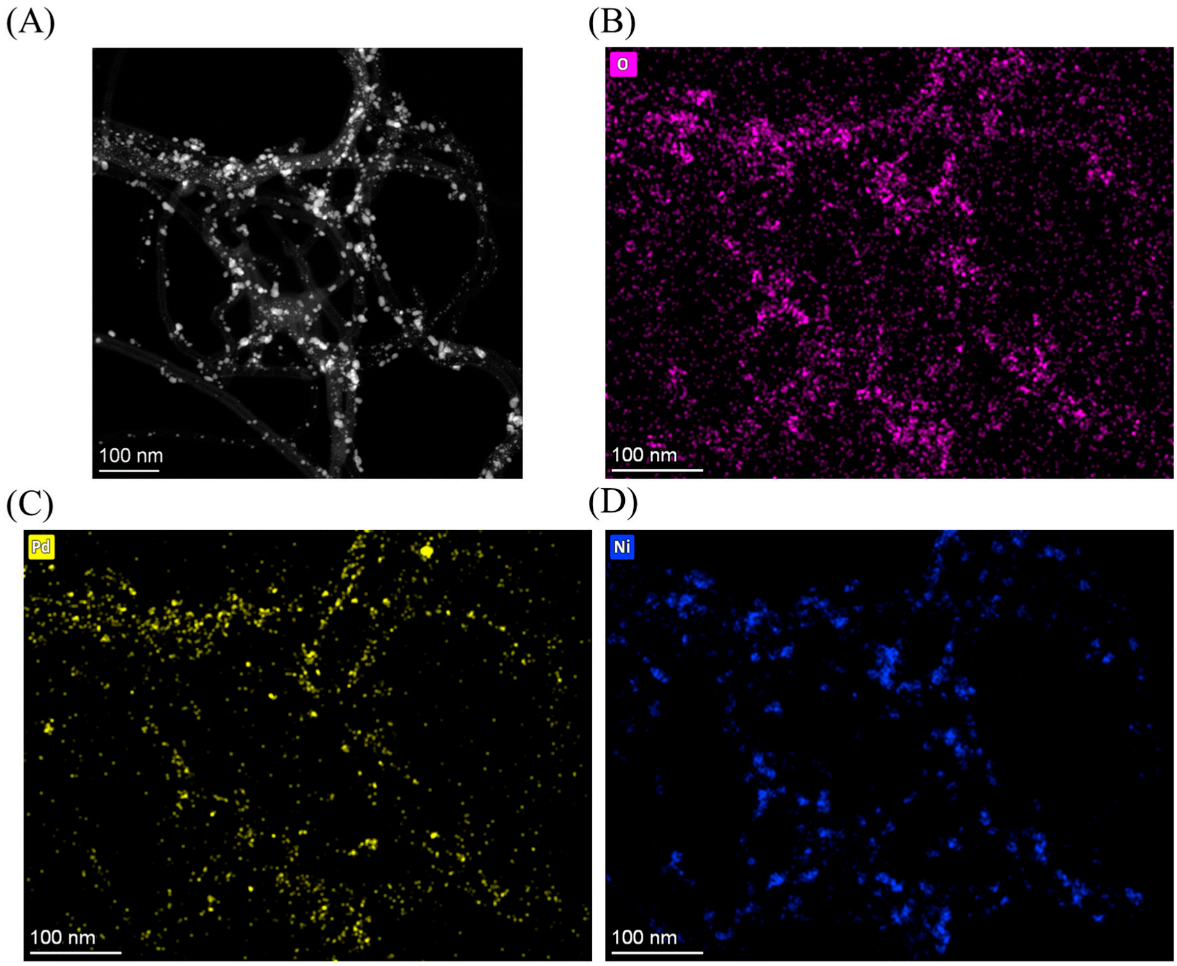

Figure S2: TEM image (A, 140000x magnification) and element mapping (B: oxygen, C: palladium, D: nickel) of $Pd_{0.33}Ni_{0.67}$/BCNT catalyst using HAADF STEM method

Comparison of the effect of 96% ethanol and absolute ethanol

We tested the effect of the 4% water content of the solvent since it could significantly improve the polarity which could be advantageous in terms of catalytic performance (50 °C, 20 barg, 0.1 g of catalyst, 0.1 M NB solution). However, the results showed that the use of absolute ethanol further increased the catalytic activity which could be explained with the excellent hydrogen solubility of the alcohol (Figure S3).

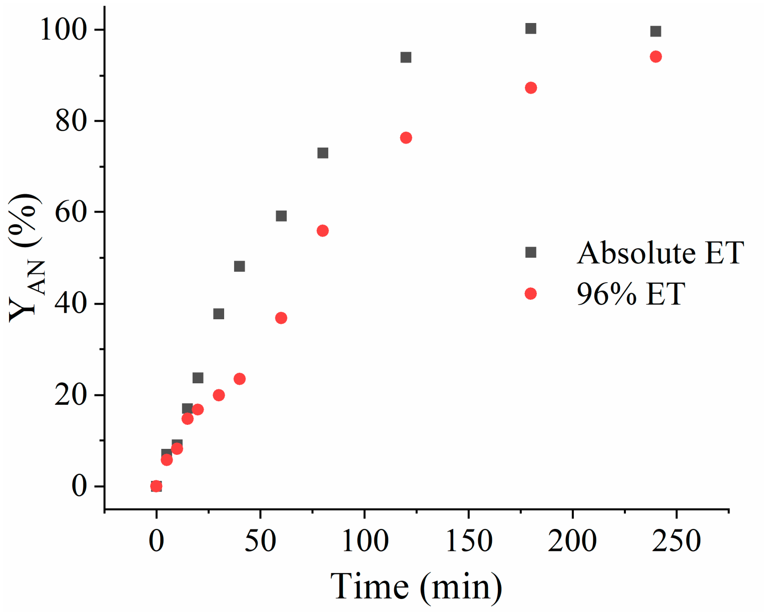

Figure S3: Comparison the use of 96% ethanol and absolute ethanol as solvent in nitrobenzene hydrogenation

Catalytic performance comparison

Turn Over Number (TON) was calculated for our and other reference catalysts for comparison [1]. TON makes it possible to compare how much product could be produced with amount of catalytic active metal on the catalyst (Eq. S1). The calculated values and the used test parameters can be seen in Table S1.

$$\boldsymbol{TON} = \frac{n_{aniline}}{n_{palladium}} \qquad \text{Eq. S1.}$$

Table S1: Comparison of the prepared $Pd_{0.33}Ni_{0.67}$/BCNT catalyst using Turn Over Number (TON) with other conventional catalysts

Reference	Catalyst	w_{cat} (g)	p (bar)	T (K)	c_{NB} (mol L^{-1})	t (min)	TON (mol_{AN}/mol_{Pd})
This paper	$Pd_{0.33}Ni_{0.67}$/BCNT	0.1	20	323	0.1	240	494.16
Prekob et al.[1]	5% Pd/CC	0.2	20	323	0.250	240	360.19
Couto et al.[2]	1% Pd/Al_2O_3	80	15	423	0.812	240	92.93
Wang et al. [3]	5.22% Pd/AC	0.025	10	313	0.326	15	295.97
Wang et al. [3]	4.24% Pd/Al_2O_3	0.025	10	313	0.326	15	77.94
Wang et al.[3]	5.28% Pd/MWCNT	0.025	10	313	0.326	15	281.09
Mironenko et al.[4]	1.5% Pd/C-PVC	0.2	5	323	0.100	30	350.47

References

1. Prekob; Hajdu, V.; Muránszky, G.; Fiser, B.; Sycheva, A.; Ferenczi, T.; Viskolcz, B.; Vanyorek, L. Application of Carbonized Cellulose-Based Catalyst in Nitrobenzene Hydrogenation. *Mater Today Chem* 2020, *17*, doi:10.1016/j.mtchem.2020.100337.
2. Couto, C.S.; Madeira, L.M.; Nunes, C.P.; Araújo, P. Hydrogenation of Nitrobenzene over a Pd/Al2O3 Catalyst - Mechanism and Effect of the Main Operating Conditions. *Chem Eng Technol* 2015, *38*, 1625–1636, doi:10.1002/ceat.201400468.
3. Wang, Z.; Liu, H.; Chen, L.; Chou, L.; Wang, X. Green and Facile Synthesis of Carbon Nanotube Supported Pd Nanoparticle Catalysts and Their Application in the Hydrogenation of Nitrobenzene. *J Mater Res* 2013, *28*, 1326–1333, doi:10.1557/jmr.2013.101.
4. Mironenko, R.M.; Belskaya, O.B.; Kryazhev, Y.G.; Gulyaeva, T.I.; Likholobov, V.A. Palladium Hydrogenation Catalysts Prepared Using Porous Carbon Materials Derived from Poly(Vinyl Chloride). In Proceedings of the AIP Conference Proceedings; American Institute of Physics Inc., August 16 2019; Vol. 2143.
